# Fall awareness behaviour and its associated factors among community dwelling older adults

**DOI:** 10.1186/s12877-021-02122-z

**Published:** 2021-04-06

**Authors:** Jing Wen Goh, Devinder Kaur Ajit Singh, Normala Mesbah, Anis Afifa Mohd Hanafi, Adlyn Farhana Azwan

**Affiliations:** Physiotherapy Programme & Center for Healthy Ageing and Wellness, Faculty of Health Sciences, Universiti Kebangsaan Malaysia, Kuala Lumpur, Malaysia

**Keywords:** Fall awareness behaviour, Fall strategy, Fall prevention, Older adults, Older people

## Abstract

**Background:**

Falls are one of the major causes of mortality and morbidity in older adults. However, despite adoption of prevention strategies, the number of falls in older adults has not declined. The aim of this study was to examine fall awareness behaviour and its associated factors among Malaysian community dwelling older adults.

**Methods:**

A total of 144 community dwelling older adults (mean age of 70.69 ± 4.3 years) participated in this study. Physical performance were assessed using timed up and go (TUG), gait speed (GS), chair stand and hand grip tests. Fall Awareness Behaviour (FaB) and Fall Risk Assessment Questionnaires (FRAQ) were administered to assess behaviour and fall prevention knowledge respectively.

**Results:**

Stepwise linear regression analysis showed that the practice of fall awareness behaviour (R^2^ = 0.256) was significantly associated with being male [95% C.I: 2.178 to 7.789, *p* < 0.001], having lower BMI [95% C.I: − 0.692 to − 0.135, *p* < 0.05], living with family [95% C.I: 0.022 to 5.953, p < 0.05] and those having higher functional mobility [95% C.I: − 2.008 to − 0.164, *p* < 0.05].

**Conclusions:**

Fall awareness behaviour should be emphasized among older females, those with lower functional mobility, higher BMI and living alone.

**Supplementary Information:**

The online version contains supplementary material available at 10.1186/s12877-021-02122-z.

## Background

Falls are a public health concern that results in increased mortality and morbidity among older adults [1]^.^ Falls can lead to devastating health consequences including injuries, hospitalization, emergency department visits and increased health care burden among older population [2]^.^ Unintentional falls have been identified as the fifth leading cause of death among older adults [3]. The overall prevalence of falls among Malaysian community dwelling older adults is estimated to be between 4.2 to 61% depending on its settings [4]. This is of a higher prevalence as compared to other Asian (17.2–36.8%) [5] and Western (27–29%) [6] population.

An increased risk of falls have been reported among older adults with multiple health related problems, including osteoporosis, low calcium levels, loss of bone mineral density, low body mass index, loss of muscle strength, visual impairments, neuromuscular disorders (Parkinson disease) and cognitive impairment (Alzheirmer’s dementia). Carelessness, lack of physical activity, wearing of inappropriate shoes and improper use of assistive devices, can intensify risk of falls [3]^.^ In addition, post-fall psychological problems, fear of falls can also place older adults at an increased risk of falling [7].

Almost one-quarter of older adults restrict themselves in daily living activities, with about 30, 45 and 24% limiting indoor, outdoor and both indoor and outdoor activities respectively [5]. This restriction may lead to further falls and disabilities. The risk is also signicantly increased among those older adults who have not modified their home environment. Strategies of falls prevention adopted and practised by older adults include being aware of falling and recognising their limitations when participating in an activity [8].

A number of factors associated with adoption of falls prevention practices among older adults have been identified in existing literature. These include sex, age, educational level, living and health status, socioeconomic status, cognitive impairment, depression, impaired mobility, falls prevention knowledge and previous experience of falls [9–11]. Out of these, Gaspar et al. (2017) [12] found that there was higher adoption of falls prevention practices among older male adults, those who have good self-rated health status and higher education levels. A significant association has also been found between age and adoption of falls prevention whereby, older adults tend to behave in a safer way with advancing age [13]. Often, older adults use assistive devices (walkers or walking sticks) to assist in their ambulation and balance, and this has led to slower and more careful movements to protect themselves from a fall [13]^.^

Despite having various falls prevention educational programmes in place, participation of older adults in falls prevention is still limited. For example, some older persons still have negative perceptions on falls prevention recommendation, different beliefs and denial of having fall risks [14]. Moreover, falls prevention practices have not been adopted among older adults globally [15]. This could possibly stem from their refusal to acknowledge their weakness and being afraid to be seen as old [16]. It is also noteworthy that most older people failed to recognize their falls risk and did not consider it as a priority and disagreed with home modifications [16]. In Malaysia, older adults are worried about the stigma of looking weak and frail and feel embarrassed about using walking aids [17].

The existing evidence suggests that falls prevention intervention should focus on falls risk factors [18, 19]. Currently, there is a lack of information regarding falls awareness and its associated factors among older adults, specific to a multicultural population such as in Malaysia. This information is essential in designing falls prevention strategies specifically tailored for personalised client centred care.

This paper sets out a hypothesis that there is an association between falls and certain preventive behaviours in relation to socio-demographic, physical and clinical factors. The aim of this present study was to examine the association between falls prevention behaviour, socio-demographic, physical and clinical factors among community-dwelling older adults. Socio-demographic factors of interest consisted of, age, sex, race, number of comorbidities, living status and education levels. Physical factors considered included mobility status, lower and upper limb muscle strength. Clinical factors examined were knowledge of falls, comorbidities, history and number of falls.

## Methods

### Study design

This descriptive and cross-sectional study was conducted in a number of districts in the state of Selangor, Malaysia, where the number of older adults are most dense, namely Tanjung Sepat, Cheras, Kajang, Dato Keramat, Petaling Jaya, Klang, Sepang, Rasa, Kuala Selangor and Sekinchan between September and November 2018. The study comprised of community-dwelling older adults aged 60 and above.

### Recruitment of participants

Participants from only one state (Selangor) representing the central region of Peninsular Malaysia, that were previously involved in longitudinal study on neuroprotective model for healthy longevity (LRGS-TUA) [20] participated in the present study. Participants in LRGS-TUA longitudinal study were recruited using multistage random sampling and the details has been reported previously [20, 21].

### Inclusion and exclusion criteria

Older adults who attended the session were screened to meet the inclusion criteria and screened for cognitive impairment using Mini Mental State Examination (MMSE) and depression using Geriatric Depression Scale (GDS-15). Inclusion criteria included older adults who had no permanent disability or impairments, were able to give consent, able to ambulate independently with or without assistive devices for at least 6 m, with scores of MMSE ≥25 and GDS-15 ≤ 5. The exclusion criteria were those who scored MMSE scores ≤24, GDS-15 ≥ 6, having recent lower limb fractures and acute illnesses.

### Data collection

Verbal and written information regarding the procedure of study were provided to all participants. Subsequently, participants signed informed consent forms prior to data collection.

### Demographic data

A face-to-face interview was conducted to obtain the sociodemographic data and medical history of participants and included questions focusing on age, race, sex, employment status, education levels, living status, number of falls within the past twelve months, self-reported medical history (hypertension, diabetes, heart disease, joint pain, incontinence, vision impairments) and medications taken.

### Outcome measures

Falls awareness was assessed using Fall Awareness Behaviour Questionnaire (FaB) [22]. The FaB is a self- rating scale used to assess the actions and behaviours that older adults usually practice to prevent falls. The scale is made of thirty items and ten subscales. The subscales are: (1) protective mobility (5 items); (2) cognitive adaptations (6 items); (3) awareness (4 items); (4) avoidance (5 items); (5) pace (2 items); (6) practical strategies (3 items); (7) being observant (1 item); (8) displacing activities (1 item); (9) changes in level (2 items) and; (10) getting to phone (1 item). For each scale, the participants were required to give a score based on four categories, never, sometimes, often and always. The data was analysed based on its total score and scores could range from 30 (risky fall behaviour) to 120 (safest falls prevention behaviour). The lower the score, the more likely a person would engage in risky behaviours. Higher scores indicated a person who was more likely to be aware of falls prevention. FaB was found to have a high validity with internal consistency of 0.84 and content validity index of 0.93 [22]. The scale has been adapted for Malaysian culture in a previous local study. For the adapted scale, the Cronbach’s Alpha coefficients was 0.723 with the removal of two items, indicating an acceptable internal consistency.

Fall Risk Assesment Questionnaire (FRAQ) was used to assess falls prevention knowledge among older adults [23]. It consists of twenty-two questions and these questions evaluate falls prevention knowledge based on the apsects of: (1) behaviours (eight items); (2) environmental (five items); (3) medical condition (six items) and; (4) medication (three items). The total full score for this questionnaire is 21. Higher score suggests higher knowledge of falls among older adults [24]. FRAQ was found to have a strong agreement with clinical evaluation (kappa = 0.875, *p* < 0.001) and good validity. The kappa value for individual items is ranged from 0.305 to 0.832 [24]. The Malay version of FRAQ used in this study was one that had been translated for a previous local sudy. Its internal consistency had a Cronbach’s alpha value of 0.748 after removing one item.

Participants performed timed-up and go (TUG) test to assess balance and mobility status [25]. TUG test was found to have high sensitivty and specifity value of 87% [26]. The time taken from standing up from an armless chair with 46 cm height, walking 3 m towards a cone at their usual and comfortable pace, turning, walking back towards the chair and sitting down was recorded in seconds. The participants were instructed to wear their shoes and were allowed to walk with or without assistive devices. Time taken to complete the test was recorded in seconds.

Six metre gait speed was performed by participants as a measure of functional mobility. Time taken to complete walking at their own pace for a distance of six meters was taken in seconds.

30 s chair stand test was used to measure lower limb muscle strength among the older adults. A 17 in. (43.2 cm) plastic chair without armrests was placed against the wall to prevent the chair from slipping during the test. Participants were required to sit with their backs against the backrest, arms crossed against their chest, feet with shoulder distance apart and placed firmly on the floor. Participants were instructed to stand and sit as many times as possible within 30 s. One repetition was considered as a complete sit and stand. Chair stand test has been shown to have a high test-retest correlation for both men (0.84) and women (0.92) [27]. This test also has a good criterion related validity, r = 0.78 in men and r = 0.71 [27].

Dominant hand grip strength testing has been shown to have good validity with high correlation (r = 0.99, *p* < 0.001) [28]. The participants were asked to sit upright with elbow and shoulders positioned at a 90-degree angle, and forearms placed in neutral position. The participants were required to squeeze the dynamometer handle as hard as possible using their dominant hand on the command ‘start’ and sustain it for three seconds. This test was conducted twice and the maximum reading was taken as the result.

### Data analysis

Data was analysed using IBM Statistical Package for Social Sciences (SPSS) software version 23.0, IBM Corporation, United States. Statistical analysis of Alpha level 0.05 was used in all statistical tests. Distribution of all data were analysed using Kolmogorov-Smirnov, Shapiro-Wilk and other non-parametric tests. Means and standard deviations were calculated for the following variables: (1) age; (2) MMSE scores; (3) GDS-15 scores; (4) BMI; (5) FRAQ scores; (6) FaB scores; (7) TUG; (8) gait speed; (9) hand grip strength; (10) chair stand tests. While, percentage were described for the following variables: (1) sex; (2) race; (3) education status; (4) marital status; (5) living status; (6) working status; (7) number of comorbidities; (8) number of medication; (9) fall history.

Stepwise linear regression analysis with level at Alpha level 0.05 was used to analyse the association between the practice of falls prevention behaviour (dependent variables) and sociodemographic factors (age, sex, race, number of comorbidity, marital status, employment status, living status and education level), physical factors (mobility status, lower limb and upper limb muscle strength) and clinical factors (knowledge of falls, comorbidity, history of falls and number of falls).

### Ethics approval and consent to participate

This study was approved by the Medical Research and Ethics Committee of Universiti Kebangsaan Malaysia (UKM PPI/111/8/JEP-2018-559).

## Results

Table 1 depicts comparison of sociodemographic data among community dwelling older adults categorised as fallers and non-fallers.

**Table 1:** Sociodemographic of participants categorized based on fallers and non-fallers

Variables	Total Participants (*n* = 144)	Fallers (*n* = 31; 21.5%)	Non-fallers (*n* = 113; 78.5%)	*P* value
*Sociodemographic profiles*				
Age; mean (SD)	70.69 (4.3)	71.45 (4.6)	70.49 (4.2)	0.27
MMSE; mean (SD)	27.88 (1.6)	27.71 (1.4)	27.92 (1.7)	0.53
GDS; mean (SD)	1.49 (1.5)	1.58 (1.5)	1.47 (1.5)	0.71
Sex; n (%)				0.03*
Male	71 (49.3)	10 (14.1)	61 (85.9)	
Female	73 (50.7)	21 (28.8)	52 (71.2)	
Race; n (%)				0.15
Malay	65 (45.1)	10 (15.4)	55 (84.6)	
Chinese	60 (41.7)	16 (26.7)	44 (73.3)	
India	19 (13.2)	5 (26.3)	14 (73.7)	
Education level; n (%)				0.28
No education	8 (5.6)	3 (37.5)	5 (62.5)	
Primary	40 (27.8)	9 (22.5)	31 (77.5)	
Secondary	70 (48.6)	14 (20.0)	56 (80.8)	
Tertiary	24 (16.7)	5 (20.8)	19 (79.2)	
Others	2 (1.4)	0	2 (100)	
Marital status; n (%)				0.81
Single	2 (1.4)	1 (50.0)	1 (50.0)	
Married	112 (77.8)	23 (20.5)	89 (79.5)	
Divorce	2 (1.4)	0	2 (100.0)	
Others	28 (19.4)	7 (25.0)	21 (75.0)	
Working status; n (%)				0.20
Not working/ housewife	64 (44.4)	17 (26.6)	47 (73.4)	
Retired	63 (43.8)	11 (17.5)	52 (82.5)	
Retired but working	5 (3.5)	2 (40.0)	3 (60.0)	
Working	12 (8.3)	1 (8.3)	11 (91.7)	
Living status; n (%)				0.29
Living alone	4 (2.8)	2 (50.0)	2 (50.0)	
Living with husband/ wife	46 (31.9)	12 (26.1)	34 (73.9)	
Living with husband/ wife/ child/ grandchild	88 (61.1)	15 (17.0)	73 (83.0)	
Others	6 (4.2)	2 (33.3)	4 (66.7)	
BMI (kg/m^2^); mean (SD)	26.30 (5.29)	25.26 (4.5)	26.62 (5.5)	0.20
*Clinical factors*				
No of comorbidity; n (%)				0.09
No comorbidity	4 (2.8)	0	4 (100.0)	
One comorbidity	19 (13.2)	2 (10.5)	17 (89.5)	
≥ 2 comorbidities	121 (84.0)	29 (24.0)	92 (76.0)	
No of medication; n (%)				0.46
< 3 medication	106 (73.6)	20 (18.9)	86 (81.1)	
≥ 3 medication	38 (26.4)	11 (28.9)	27 (71.1)	
Falls history in past 12 months; n (%)				0.001*
No falls	113 (78.5)	0	113 (100)	
≥ One fall	31 (21.5)	31 (100)	0	
*Falls knowledge*				
FRAQ; mean (SD)	12.30 (3.0)	12.10 (3.3)	12.35 (2.9)	0.67
*Falls behaviours*				
FaB; mean (SD)	53.30 (9.5)	50.77 (7.7)	53.97 (9.9)	0.10
*Physical test*				
Timed up and go test (sec); mean (SD)	10.17 (1.6)	10.41 (1.9)	10.10 (1.5)	0.34
6 m Gait speed test (m/ sec); mean (SD)	1.12 (0.7)	1.29 (1.4)	1.07 (0.2)	0.42
30 s Chair stand test; mean (SD)	11.4 (2.4)	11.74 (2.9)	11.30 (2.3)	0.37
Dominant Hand grip strength test (kg); mean (SD)	21.37 (6.8)	19.61 (6.4)	21.85 (6.9)	0.11

*p < 0.05, Independent t-test

Fallers were defined as older adults who had experienced falls in past 12 months, while non-fallers were those who had no falls within the past year. Twenty-two percent of the participants had a history of a fall or falls within the past year. There was no significant difference (*p > 0.05*) between fallers and non-faller groups, except for sex. A majority of participants who were classified as fallers were females (30%) *(p = 0.03)*. There is no significant differences between the groups, but faller group had lower mean scores for FaB (50.77 ± 7.7) and FRAQ (12.10 ± 3.3) compared to the non-faller group (FaB = 53.97 ± 9.9; FRAQ = 12.35 ± 2.9) (*p* > 0.05).

Based on total FaB scores analysis, sex, BMI category, comorbidity and physical mobility (TUG test) were shown to be significantly different (*p* < 0.05) (Table 2).

**Table 2:** Comparison of sociodemographic profiles, clinical and physical factors according to subscale scores of FaB

Variables	Total score; mean ± SD	Cognitive adaptation; mean ± SD	Protective mobility; mean ± SD	Avoidance; mean ± SD	Awareness; mean ± SD	Pace; mean ± SD	Practical strategies; mean ± SD	Displacing activities; mean ± SD	Being observant; mean ± SD	Changes in level; mean ± SD	Getting to the phone; mean ± SD
*Sociodemographic profiles*											
Age											
65-69;n (67)	53.91±9.01	10.00±3.32	10.78±3.73	10.00±3.20	6.63±2.76	5.21±1.57	7.72±2.32	3.30±1.00	1.48±0.99	2.37±1.51	1.69±1.06
70-74;n (48)	53.19±10.79	9.75±3.47	10.44±3.43	9.38±2.51	6.77±2.82	5.71±1.50	7.65±2.58	3.19±1.14	1.35±0.86	2.46±1.34	2.08±1.22
75-79;n (21)	50.41±6.54	8.81±3.36	9.38±2.78	9.71±2.95	6.19±2.04	5.81±1.36	7.48±1.89	3.19±1.25	1.38±0.74	1.71±0.64	2.14±1.24
80-84;n (8)	56.13±11.74	11.5±3.89	11.88±4.40	9.25±3.11	6.38±2.26	5.38±0.52	7.13±2.36	3.00±1.07	2.00±1.20	2.75±1.91	2.00±1.07
Gender											
Male;n (71)	56.13±10.35	10.97±3.66	10.63±3.88	10.79±2.90	6.83±2.72	5.42±1.42	8.08±2.49	3.34±1.04	1.58±1.08	2.44±1.34	1.89±1.12
Female;n (73)	50.51±7.71***	8.71±2.77***	10.41±3.23	8.66±2.56***	6.37±2.57	5.52±1.56	7.18±2.10*	3.12±1.12	1.33±0.75	2.22±1.45	1.92±1.19
Education level											
No education;n (8)	50.18±7.63	8.13±3.87	10.88±5.00	8.38±1.92	5.88±2.17	5.00±0.93	7.13±2.3	3.75±0.46	1.25±0.71	1.63±0.92	2.50±1.41
Primary;n (40)	56.31±7.22	10.60±2.92	12.40±3.18	9.38±2.52	7.15±2.37	5.65±1.76	8.08±2.52	2.95±1.20	1.75±1.15	2.18±1.41	1.70±1.11
Secondary;n (70)	51.33±10.14	9.41±3.47	9.57±3.35	9.46±2.91	6.30±2.83	5.40±1.46	7.16±2.24	3.29±1.07	1.37±0.80	2.33±1.29	2.06±1.19
Tertiary;n (24)	54.44±10.50	10.04±3.74	9.92±3.16	11.08±3.31	6.83±2.73	5.58±1.32	8.42±2.15	3.42±0.93	1.17±0.70	2.79±1.67	1.63±0.92
Others;n (2)	59.53±6.16	113.00±1.41	12.00±4.24***	14.00±2.83**	6.00	5.00	7.5±2.12	2.50±2.12	2.50±2.12*	2.50±2.12	1.50±0.71
Living status											
Living alone;n (4)	47.40±6.91	6.25±2.99	8.50±2.65	8.75±3.59	6.75±2.63	5.75±1.71	6.50±1.29	3.25±1.50	1.00	2.00±0.82	1.75±1.50
Living with husband/ wife;n (46)	51.59±9.87	9.50±3.29	9.70±3.45	9.57±2.90	6.41±2.48	5.52±1.35	7.48±2.18	3.35±0.99	1.46±0.96	2.33±1.28	1.91±1.17
Living with husband/ wife/ child/ grandchild;n (88)	54.41±9.40	10.09±3.39	11.11±3.63	9.83±2.96	6.65±2.74	5.38±1.56	7.75±2.48	3.17±1.11	1.50±0.96	2.32±1.47	1.94±1.15
Others;n (6)	53.53±8.12	10.83±4.26	9.50±2.17	9.67±2.66	7.17±3.06	6.33±1.37	7.67±1.97	3.17±1.33	1.00	2.67±1.51	1.33±0.82
BMI											
Under weight;n (5)	65.71±11.95	12.80±5.54	13.60±3.21	13.20±2.95	6.60±3.78	5.40±0.55	10.40±1.82	3.20±1.30	1.20±0.45	3.60±2.07	2.40±1.52
Normal weight;n (58)	55.38±9.33	10.34±3.38	11.00±3.83	10.36±2.84	6.57±2.51	5.66±1.49	7.78±2.35	3.29±1.12	1.52±0.94	2.34±1.29	1.95±1.15
Overweight;n (53)	51.84±8.55	9.38±3.47	10.26±3.20	9.17±2.76	6.45±2.58	5.53±1.41	7.47±2.37	3.25±1.02	1.51±0.99	2.13±1.23	1.81±1.14
Obese;n (28)	49.44±8.68***	9.07±2.60	9.46±3.37	8.75±2.77**	6.93±2.94	5.00±1.70	7.11±2.04*	3.07±1.12	1.25±0.84	2.43±1.69	1.89±1.13
*Clinical factors*											
No of comorbidity											
No comorbidity;n (4)	63.00±12.79	12.50±3.70	13.25±2.22	8.75±1.71	8.00±2.16	6.50±1.29	9.75±2.22	2.50±1.73	2.50±1.29	3.50±1.73	2.25±1.26
One comorbidity;n (19)	56.46±12.90	11.32±3.71	10.74±4.41	10.95±3.21	4.95±3.12	5.47±1.84	7.79±2.82	3.53±0.70	1.68±1.06	2.21±1.58	2.00±1.16
≥ 2 comorbidities;n (121)	52.46±8.56*	9.50±3.29*	10.40±3.43	9.55±2.88	6.81±2.49**	5.44±1.44	7.53±2.24	3.21±1.10	1.39±0.88*	2.31±1.35	1.88±1.15
No of medication											
< 3 medication;n (106)	53.36±9.39	9.82±3.46	10.58±3.72	9.73±2.85	6.48±2.68	5.37±1.46	7.54±2.31	3.24±1.07	1.49±0.96	2.36±1.44	1.90±1.15
≥ 3 medication;n (38)	53.05±9.93	9.84±3.36	10.37±3.11	9.66±3.17	6.92±2.54	5.76±1.57	7.87±2.43	3.21±1.14	1.34±0.85	2.24±1.28	1.92±1.15
Falls history in past 12 months											
No falls;n (31)	50.77±7.75	9.16±3.18	9.65±3.50	8.84±2.79	6.03±2.12	5.61±1.33	7.48±2.05	3.23±1.06	1.35±0.80	2.23±1.36	2.03±1.17
≥ One falls;n (113)	53.97±9.85	10.01±3.47	10.76±3.55	9.95±2.93	6.75±2.76	5.43±1.53	7.66±2.42	3.23±1.09	1.48±0.97	2.35±1.41	1.87±1.15
*Physical test*											
TUG test											
<11.18s;n (108)	54.31±9.50	10.09±3.48	10.88±3.41	9.99±3.06	6.61±2.77	5.55±1.42	7.75±2.32	3.23±1.10	1.48±0.96	2.44±1.48	1.88±1.15
≥11.18s;n (36)	50.19±8.94*	9.03±3.14	9.44±3.81*	8.86±2.29*	6.56±2.25	5.25±1.68	7.25±2.39	3.22±1.05	1.36±0.83	1.97±1.03*	1.97±1.16

*p < 0.05, **p < 0.01, ***p < 0.001,Independent t-test, One-way ANOVA

The total FaB scores were significantly higher among males (56.13 ± 10.35) compared to females (50.51 ± 7.71) (*p* < 0.05). While the TUG cut-off used in our study was 11.18 s [29], older adults who took less time to complete the TUG test (54.31 ± 9.50) scored significantly higher in FaB as compared to those who took a longer time to complete the TUG test (50.19 ± 8.94) (*p* < 0.05). Post hoc analysis (using an α of 0.05) for BMI group and comorbidity were conducted. The analysis revealed that older adults who were underweight (65.71 ± 11.95) had significantly higher FaB scores than those who were of normal weight (55.38 ± 9.33), overweight (51.84 ± 8.55) and obese (49.44 ± 8.68) (*p* < 0.05). However, there was no significant difference between FaB scores of older adults who were normal weight and overweight (*p* > 0.05), nor between the FaB scores of those who were overweight and obese (*p* > 0.05). Post hoc analysis results also showed that there were significantly higher scores of FAB between those without comorbidity (63.00 ± 12.79) and with more than two comorbidities (52.46 ± 8.56) (*p* < 0.05).

When the sociodemographic profiles, clinical factors and physical tests were assessed according to FaB subscales, significant differences were identified in the subscales of *cognitive adaptation, protective mobility, awareness, avoidance, practical strategies, being observant* and *changes in level* (*p* < 0.05). Males obtained significantly higher scores for *cognitive adaptation* (*p* < 0.001), *avoidance* (*p* < 0.001) and *practical strategies* (*p* < 0.05) subscales compared to females. The participants with primary education were found to have a better score for *protective mobility* subscales (*p* < 0.001) compared to those without any education, secondary or tertiary. In addition, the *avoidance* (*p* < 0.05) and *practical strategies* (*p* < 0.05) subscales were also significantly higher among older adults who were underweight. In terms of clinical factors, participants without any comorbidity were reported to score significantly higher in *cognitive adaptation* (*p* < 0.05), *awareness* (*p* < 0.05) and *being observant* (*p* < 0.05) subscales. Participants who took less than 11.18 s to complete TUG test achieved significantly higher scores for *protective mobility* (*p* < 0.05), *avoidance* (*p* < 0.05) and *changes in level* (*p* < 0.05) subscales in comparison to those who took a longer time to complete TUG test.

Stepwise linear regression results of the association between sociodemographic profiles, clinical and physical factors, falls knowledge and awareness, and the practice of its prevention behaviour *(R*^*2*^ *= 0.256)* are as depicted in Table 3.

**Table 3:** Linear regression (Stepwise) model: Variables associated with fall prevention practices among older adults

Variables	Unstandardized Coefficients	Standardized Coefficients	95% C.I for Beta		Collinearity Statistics	*P*- value	R^2^
	*B(S)*	Beta	Lower	Upper	Tolerance		
Male	4.984 (1.419)	0.263	2.178	7.789	0.961	0.001*	0.256
TUG Test	−1.086 (0.466)	−0.182	−2.008	−0.164	0.881	0.02*	
No. of comorbidity	−0.625 (0.348)	−0.141	−1.313	0.64	0.871	0.075	
BMI (mean)	−0.414 (0.141)	−0.230	−0.692	−0.135	0.876	0.004*	
Living with family (husband,/wife and children	2.988 (1.5)	0.154	0.022	5.953	0.905	0.05*	

Linear Reggresion (Stepwise) test of association*p < =0.05. Excluded variables: Race, Age, Years of education, Working status, Hand Grip test, Chair stand test, Gait speed test, History of fall, No. of fall, Total FRAQ

When assessed based on the FaB total mean score, older males [95% C.I: 2.178 to 7.789, *p* < 0.001] and those living with family [95% C.I: 0.022 to 5.953, *p* < 0.05] were found to have higher scores in practice of falls prevention behaviours compared to females and those living alone. Mean BMI [95% C.I: − 0.692 to − 0.135, *p* < 0.05] and TUG test [95% C.I: − 2.008 to − 0.164, *p* < 0.05] were inversely proportional to FaB scores, indicating participants with lower BMI and took less time to complete the TUG test had higher falls prevention practice scores. Although not statistically significant (*p* > 0.05), the participants with fewer comorbidities were more likely to anticipate these prevention behaviours actively [95% C.I: − 1.313 to − 0.64, *p* > 0.05].

## Discussion

Prevention of falls awareness and its practice is of utmost importance. The aim of our study was to determine the sociodemographic, clinical and physical factors associated with falls awareness and falls prevention behaviour among community dwelling older adults. In our study, we found that there was a significant association between the practice of falls prevention behaviour with males, having lower BMI, higher functional mobility and living with family in Malaysian based community dwelling older adults.

Falls prevention behaviours were significantly associated with male older adults having higher prevalance of falls prevention practice, specifically *cognitive adaptation*, *avoidance* and *practical strategies* subscales compared to females. This result is consistent with a study by Gaspar et al. (2017) [12], where males were shown to be more active in falls prevention practices compared to females. Similarly, a moderate to strong significant association was found between males actively involved in self-management of falls prevention behaviour in another recent study [30].

Moreover, it was shown that there is greater adherence in falls prevention programmes among males compared to female older adults [9]^.^ Although females were reported to be more active in seeking medical attention after a fall or to gather more information about falls prevention compared to males, they were more inclined to limit their daily activities and physical function due to fear of falls [31, 32]. We also deduced that higher falls prevention practice among male older adults in our study findings could be linked to the fact that there is a higher likelihood of falls among females estabished in the literature [33]. In addition, males were reported to be less likely to report falls or engage in falls prevention programs in comparison to females [34]. However, further studies regarding falls prevention practice in view of the differences between older males and females is warranted.

A relationship between higher falls prevention behaviour and living with family was demonstrated in our study. The plausible explanation for this could be that older adults living with family members are known to have more social support which empowers them to participate and engage in daily activities [35, 36]. Moreover, family members could have played a role in providing falls prevention and environmental hazard awareness besides discouraging risky behaviours among older adults [35]. In contrast, older adults who live alone practice restriction in their daily activities due to fear of falls, which may in turn reduce their engagement in falls prevention behavaior [30]. There were no significant difference in the living status between fallers and non-fallers in our present study.

It is worth noting that participants without any comorbidity were particularly more careful and aware of external fall risk factors, as they scored higher in these FaB subscales in our study. These results are consistent with findings that, participation in fall prevention behaviour was higher in older adults who actively managed their health [37]. Polypharmacy as a result of multiple comorbidities would increase risk of falls among older adults [3]^,^ thereby limiting their daily living activities to keep themselves safe. This may reduce their engagement in falls prevention practices [38].

An association between increased falls risk in older adults with higher BMI has been highlighted in literature [39]. In older adults, excessive weight may result in a reduction of muscle strength and consequently reduce physical function [40]. This may explain the results in our study whereby older adults with obesity were associated with lower involvement in falls prevention practices due to physical inability [41]. In addition, older adults with obesity have been shown to be more likely to have fatalistic views that no efforts can be taken to prevent themselves from falls [42]. Decrease in physical activity levels, postural stability and physical functional levels due to obesity could possibly compound the reason for lower engagements in overall activities [41].

In line with the results of our study, previous studies have demonstrated that independent older adults had greater interest and engagement in falls prevention practices compared to older adults who are dependent [12]^.^ Participants who were more aware about falls prevention behaviour practices took shorter time to complete TUG test in our study. This group of participants also scored higher in *changes in level* subscale, in which they had more ability to cope with the challenging activities, especially when climbing up and down stairs.

This result suggests that falls prevention practices were less common among older adults with reduced functional mobility. With deterioration of physical function, mobility and self-management ability due to reduced muscle strength and increased fear of falling, older adults were less likely to engage in falls prevention practices [43, 44]. Furthermore, older adults who are dependent have difficulty in performing a task or activity in addition to fear of falls during movements [45, 46]. Therefore, they are unable and less willing to engage actively in falls prevention practices compared to older adults with greater mobility.

While there was no significant relation between falls risk knowledge and the practice of its preventive behaviours in our study, fallers had lower mean scores of FRAQ compared to non-fallers. The practice of falls prevention among older adults could be significantly influenced by falls risk knowledge and awareness whereby higher educational levels can lead to better prevention behaviours [12]. Often, older adults with higher education levels tend to acquire more information compared to those with lower education levels [47]. With higher levels of education, older adults probably understand guidance by health care professionals, and practise falls prevention behaviour in their daily routine.

One limitation is that this study was that it was conducted among community dwelling older adults and therefore the results may not be applicable to older adults in institutions or hospitals. Moreover, this study was conducted in a single state in Malaysia. However, we used multistage random sampling methods to best represent the participants. Future large-scale studies are required to determine the factors associated with falls prevention behaviour among older adults.

## Conclusions

Our present study identified four factors associated in falls prevention behaviour practices among older adults which includes, being males, living with family, having lower BMI and higher functional mobility. Addressing these factors may be helpful in empowering at-risk older adults in falls prevention behaviours. The findings from our current study suggest that there is a need to empower older adults to engage in falls prevention behaviour, especially among women, those with high BMI, lower functional mobility and living alone.

## Supplementary Information

**Additional file 1.** Sociodemographic data.

## Data Availability

The datasets used and/or analyzed during the current study are available from the corresponding author on reasonable request.

## Abbreviations

NICE: The National Institute for Health and Care Excellence
LRGS-TUA: Longitudinal Study on Neuroprotective Model for Healthy Longevity
MMSE: Mini Mental State Examination
GDS-15: Geriatric Depression Scale-15
FaB: Fall Awareness Behaviour Questionnaire
FRAQ: Fall Risk Assessment Questionnaire
TUG: Timed-up and go
SPSS: Statistical Package for Social Sciences
BMI: Body Mass Index

## References

[1] Yamazaki Y, Hayashida CT, Yontz V (2017). Insights about Fall Prevention of Older Adults in the State of Hawai’i. Hawaii J Med Public Health.

[2] National Institute of Clinical Evidence (2017). The assessment and prevention of falls in older people. National Institute of Clinical Evidence.

[3] Ambrose AF, Paul G, Hausdorff JM (2013). Risk factors for falls among older adults: A review of the literature. Maturitas.

[4] Shaharudin MI, Kaur D, Singh A, Shahar S. FALLS PREVALENCE AND ITS RISK ASSESSMENT TOOLS AMONG MALAYSIAN COMMUNITY-DWELLING OLDER ADULTS: A REVIEW. Libk. 18. Malaysian J Public Health Med. 2018;18(2):35–38.

[5] Mane AB, Sanjana T, Patil PR, Sriniwas T (2014). Prevalence and correlates of fear of falling among elderly population in urban area of Karnataka, India. J Midlife Health.

[6] Filho JE, Borel WP, Mata Diz JB, Carvalho Barbosa AW, Britto RR, Felício DC (2019). Prevalence of falls and associated factors in community-dwelling older Brazilians: A systematic review and meta-analysis. Libk. 35, Cadernos de Saude Publica. Fundacao Oswaldo Cruz.

[7] Pitchai P, Dedhia H, Bhandari N, Krishnan D, D’Souza NJ, Bellara J (2019). Prevalence, risk factors, circumstances for falls and level of functional independence among geriatric population - A descriptive study. Indian J Public Health.

[8] Uymaz PE, Nahcivan NO (2015). Evaluation of a nurse-led fall prevention education program in Turkish nursing home residents. Educ Gerontol.

[9] Stineman MG, Strumpf N, Kurichi JE, Charles J, Grisso JA, Jayadevappa R (2011). Attempts to reach the oldest and frailest: recruitment, adherence, and retention of urban elderly persons to a falls reduction exercise program. Gerontologist.

[10] Dickinson A, Horton K, Machen I, Bunn F, Cove J, Jain D (2011). The role of health professionals in promoting the uptake of fall prevention interventions: a qualitative study of older people’s views. Age Ageing.

[11] Merom D, Pye V, Macniven R, van der Ploeg H, Milat A, Sherrington C (2012). Prevalence and correlates of participation in fall prevention exercise/physical activity by older adults. Prev Med (Baltim).

[12] Gaspar ACM, de S Azevedo RC, AAO R, Mendes PA, Segri NJ, ACM G, et al. Factors associated with fall prevention practices in older adults. Esc Anna Nery Rev Enferm. 2017;21(2):20170044.

[13] Bilik O, Turhan Damar H, Karayurt O, Damar TH (2017). Fall behaviors and risk factors among elderly patients with hip fractures. Acta Paul Enferm.

[14] Florence CS, Bergen G, Atherly A, Burns E, Stevens J, Drake C (2018). Medical Costs of Fatal and Nonfatal Falls in Older Adults. J Am Geriatr Soc.

[15] Gopaul K, Connelly DM (2012). Fall Risk Beliefs and Behaviors Following a Fall in Community-Dwelling Older Adults: A Pilot Study. Phys Occup Ther Geriatr.

[16] Stevens JA, Sleet DA, Rubenstein LZ (2018). The Influence of Older Adults’ Beliefs and Attitudes on Adopting Fall Prevention Behaviors. Am J Lifestyle Med.

[17] Loganathan A, Ng CJ, Low WY (2016). Views and experiences of Malaysian older persons about falls and their prevention-A qualitative study. BMC Geriatr.

[18] Vivrette RL, Rubenstein LZ, Martin JL, Josephson KR, Kramer BJ (2011). Development of a fall-risk self-assessment for community-dwelling seniors. J Aging Phys Act.

[19] Tricco AC, Thomas SM, Veroniki AA, Hamid JS, Cogo E, Strifler L, et al. Comparisons of Interventions for Preventing Falls in Older Adults. JAMA. 2017;318(17):1687. 10.1001/jama.2017.15006 PMC5818787 29114830

[20] Shahar S, Omar A, Vanoh D, Hamid TA, Mukari SZMS, Din NC, et al. Approaches in methodology for population-based longitudinal study on neuroprotective model for healthy longevity (TUA) among Malaysian Older Adults. Aging Clin Exp Res. 2015;28(6):1089–1104. 10.1007/s40520-015-0511-4 26670602

[21] Lau H, Shahar S, Hussin N, Kamarudin MZA, Hamid TAT, Mukari SZMS (2019). Methodology approaches and challenges in population-based longitudinal study of a neuroprotective model for healthy longevity. Geriatr Gerontol Int.

[22] Clemson L, Cumming RG, Heard R (2003). The development of an assessment to evaluate behavioral factors associated with falling. Am J Occup Ther.

[23] Sadowski C, Jones A, Feeny D (2006). The Falls Risk Awareness Questionnaire: development and validation for use with older adults. Article in Journal of Gerontological Nursing.

[24] Rubenstein LZ, Vivrette R, Harker JO, Stevens JA, Kramer BJ (2011). Validating an evidence-based, self-rated fall risk questionnaire (FRQ) for older adults. J Safety Res.

[25] Podsiadlo D, Richardson S (1991). The Timed “Up &amp; Go”: A Test of Basic Functional Mobility for Frail Elderly Persons. J Am Geriatr Soc.

[26] Shumway-Cook A, Brauer S, Woollacott M (2000). Predicting the probability for falls in community-dwelling older adults using the Timed Up &amp; Go Test. Phys Ther.

[27] Jones CJ, Rikli RE, Beam WC (1999). A 30-s Chair-Stand Test as a Measure of Lower Body Strength in Community-Residing Older Adults. Res Q Exerc Sport.

[28] de Dobbeleer L, Beyer I, Hansen ÅM, Molbo D, Mortensen EL, Lund R (2019). Grip Work Measurement with the Jamar Dynamometer: Validation of a Simple Equation for Clinical Use. J Nutr Health Aging.

[29] Ibrahim A, Singh DKA, Shahar S (2017). ‘Timed Up and Go’ test: Age, gender and cognitive impairment stratified normative values of older adults. Ginsberg SD, argitaratzailea. PLoS One.

[30] Schnock KO, Dykes PC, P. Howard E (2019). Fall Prevention Self-Management Among Older Adults: A Systematic Review. Am J Prev Med.

[31] Batra A, Page T, Melchior M, Seff L, Vieira ER, Palmer RC (2013). Factors associated with the completion of falls prevention program. Health Educ Res.

[32] Stevens JA, Noonan RK, Rubenstein LZ (2010). Older Adult Fall Prevention: Perceptions, Beliefs, and Behaviors. Am J Lifestyle Med.

[33] Sousa LMM, Marques-Vieira CMA, CMAD H, SSP S, SMA C, Caldevilla MNGN de (2017). Risk for falls among community-dwelling older people: systematic literature review. Libk. 37, Revista gaucha de enfermagem.

[34] Stevens JA, Ballesteros MF, Mack KA, Rudd RA, DeCaro E, Adler G (2012). Gender differences in seeking care for falls in the aged medicare population. Am J Prev Med.

[35] Durbin L, Kharrazi RJ, Graber R, Mielenz TJ. Social support and older adult falls. Inj Epidemiol. 2016;3(1):4. 10.1186/s40621-016-0070-y PMC4744831 27747541

[36] Elliott S, Painter J, Hudson S (2009). Living Alone and Fall Risk Factors in Community-Dwelling Middle Age and Older Adults. J Community Health.

[37] Kiyoshi-Teo H, Northrup-Snyder K, Cohen DJ, Dieckmann N, Stoyles S, Winters-Stone K (2019). Older hospital inpatients’ fall risk factors, perceptions, and daily activities to prevent falling. Geriatr Nurs (Minneap).

[38] Casteel C, Jones J, Gildner P, Bowling JM, Blalock SJ (2018). Falls Risks and Prevention Behaviors Among Community-Dwelling Homebound and Non-Homebound Older Adults. J Appl Gerontol.

[39] G. R. Neri S, Oliveira JS, B. Dario A, M. lima R, Tiedemann A. Does obesity increase the risk and severity of falls in people aged 60 years and older? A systematic review and meta-analysis of observational studies. J Gerontol Ser A. 2019. 10.1093/gerona/glz272 31750880

[40] Kalyani RR, Corriere M, Ferrucci L (2014). Age-related and disease-related muscle loss: the effect of diabetes, obesity, and other diseases. Lancet Diabetes Endocrinol.

[41] Fjeldstad C, Fjeldstad AS, Acree LS, Nickel KJ, Gardner AW (2008). The influence of obesity on falls and quality of life. Dyn Med.

[42] Mitchell RJ, Lord SR, Harvey LA, Close JCT (2014). Associations between obesity and overweight and fall risk, health status and quality of life in older people. Aust N Z J Public Health.

[43] Lee S, Lee Y-S, Kim J. Automated Evaluation of Upper-limb Motor Function Impairment using Fugl-Meyer Assessment. IEEE. 2017;26(1):125–34. 10.1109/TNSRE.2017.2755667 28952944

[44] Olij BF, Barmentloo LM, Smilde D, van der Velde N, Polinder S, Schoon Y, et al. Factors Associated with Participation of Community-Dwelling Older Adults in a Home-Based Falls Prevention Program. Int J Environ Res Public Health. 2019;16(6):1087. 10.3390/ijerph16061087 PMC6466058 30917618

[45] Lee J, Choi M, Kim CO (2017). Falls, a fear of falling and related factors in older adults with complex chronic disease. J Clin Nurs.

[46] Cawthon PM, Orwoll ES, Peters KE, Ensrud KE, Cauley JA, Kado DM (2019). Strong Relation Between Muscle Mass Determined by D3-creatine Dilution, Physical Performance, and Incidence of Falls and Mobility Limitations in a Prospective Cohort of Older Men. J Gerontol Ser A.

[47] Feinberg I, Frijters J, Johnson-Lawrence V, Greenberg D, Nightingale E, Moodie C. Examining Associations between health information seeking behavior and adult education status in the U.S.: An analysis of the 2012 PIAAC data. PLoS One. 2016;11(2):e0148751. 10.1371/journal.pone.0148751 PMC4755661 26882339

