# Supplementary material for: Fall awareness behaviour and its associated factors among community dwelling older adults
Source: BMC Geriatr. 2021 Apr 6;21:226. doi: 10.1186/s12877-021-02122-z (PMC8022521; doi:10.1186/s12877-021-02122-z)
Supplement: Supplementary file 1 — Additional file 1. Sociodemographic data. [file 12877_2021_2122_MOESM1_ESM.docx]

**DEMOGRAPHIC DATA SHEET/ *DATA DEMOGRAPIK PESERTA***

**GENERAL INFORMATION/** *MAKLUMAT UMUM*

| **Name of Participant/** *Nama Peserta* |  |
| --- | --- |
| **Participant’s Id/** *No. Id Peserta* |  |
| **IC Number/** *No Kad Pengenalan* |  |
| **Age/** *Umur* |  |
| **Gender/** *Jantina* |  |
| **Race/** *Bangsa* |  |
| **Date of Birth/** *Tarikh Lahir* |  |
| **Home Address/** *Alamat Rumah* |  |
| **Contact Number/** *No. Tel* |  |
| **Contact Number Care giver (Children or others)/** *No. Tel Penjaga (Anak atau lain)* |  |
| **Email/** *Emel* |  |
| **Education Level/** *Peringkat Pendidikan* | Primary  Secondary  Tertiary |
| **Date of Initial Assessment of FallSA/** *Tarikh Ujian Awal FallSA* |  |
| **Use of Assistive Devices/** *Penggunaan Alat Bantuan* |  |
| **FallSA Average of Physical Performance test/**  *Purata Ujian Keupayaan Fizikal* |  |
| **Any falls in past 12 months/**  *Sejarah insiden jatuh dalam masa 12 bulan yang lalu* |  |
| **Fear of falling/** *Perasaan Takut Jatuh* |  |
| **Knee pain/** *Sakit lutut* |  |
| **Eye impairment/** *Masalah Mata* |  |
| **FallSA Fall Risk Score/** *Skor Risiko Jatuh* |  |

**Falls History/** *Sejarah Insiden Jatuh*

| **Any falls in past 12 months/**  *Sejarah insiden jatuh dalam masa 12 bulan yang lalu:* | **YES/** *YA*  **NO/** *TIDAK* |
| --- | --- |
| **If yes, please state the number of falls in past 12 months/**  *Jika ya, sila nyatakan berapa kali anda terjatuhdalam masa 12 bulan yang terdekat:* |  |
| **If yes, please state the place of falls/**  *Jika ya, sila nyatakan tempat anda terjatuh:* | **Room/** *Bilik*  **Bathroom/** *Bilik Air*  **Stairs/** *Tangga*  **Outside the house/**  *Luar Rumah*  **Others/** *Lain-lain***:**  **………………………………….** |
| **If yes, please state the causes of falls/**  *Jika ya, sila nyatakan sebab anda terjatuh:* | **Tripping/** *Tersadung*  **Slipping/** *Tergelincir*  **Loss Balance/**  *Hilang Kesimbangan*  **Dizziness/** *Pening*  **Others/** *Lain-lain***:**  **........................................................** |
| **How do you getting up after falls?/**  *Macam mana anda bangun selepas jatuh?* | **Getting up by youself/**  *Bangun sendiri*  **If yes, please state how long you took to get up after falls/**  *Jika ya, sila nyatakan berapa lama anda gunakan untuk bangun selepas jatuh:*  **........................................................**  **Asking help from**  **someone/** *Minta tolong*  *daripada orang lain*  **If yes, please state how long you took to get up after falls with the help from others/**  *Jika ya, sila nyatakan berapa lama anda gunakan untuk bangun selepas jatuh dengan bantuan orang lain:*  ***........................................................*** |
| **Any injury after falls?/**  *Mengalami kecederaan setelah jatuh?* | **YES/** *YA*  **NO/** *TIDAK* |
| **If yes, any treatment done following the injury?/**  *Jika ya, adakah anda menjalani rawatan selepas jatuh?* | **YES/** *YA*  **NO/** *TIDAK*  **If yes, please state what treatment have been done following the injury?/**  *Jika ya,sila nyatakan apakah rawatan yang telah dijalani?*  **........................................................** |
| **Have you had any falls history before this past one year?**  *Adakah anda mengalami insiden jatuh sebelum satu tahun ini?* | **YES/** *YA*  **NO/** *TIDAK* |
| **Use of walking aids**  *Penggunaan peralatan bantuan jalan /tongkat:* | **YES/** *YA*  **NO/** *TIDAK*  **If yes, please state the types of walking aids/**  *Jika ya, sila nyatakan jenis :*  **........................................................** |

| **Do you present with any chronic medical illness or health problems, hypertension, heart disease, respiratory disease and neurological problems, such as stroke, dementia or Parkinson?/**  *Adakah anda mempunyai penyakit kronik, tekanan darah tinggi, penyakit jantung, penyakit peparu dan masalah neurological, seperti stroke, dementia atau Parkinson?* | **YES/** *YA*  **NO/** *TIDAK*  **If yes, please state**/ *Jika ada, sila nyatakan:*  **..........................................................** |
| --- | --- |
| **Are you taking any medication?/**  *Adakah anda mengambil ubat sekarang?* | **YES/** *YA*  **NO/** *TIDAK*  **If yes, please state***/ Jika ada, sila nyatakan:*   1. **The type**/ *Jenis*:   **...........................................................**   1. **Number of medication**/ *Jumlah ubatan:*   **...........................................................** |
| **Do you have current or recent ear infection or vestibular disorder? (in past 1 month)/**  *Adakah anda menghadapi jangkitan telinga atau masalah vestibular sekarang atau kebelakangan ini? (dalam tempoh 1 bulan)* | **YES/** *YA*  **NO/** *TIDAK* |
| **Do you have current or recent eyes problem and having blurring vision?/**  *Adakah anda menghadapi masalah mata dan penglihatan sekarang atau kebelakangan ini?* | **YES/** *YA*  **NO/** *TIDAK* |
